# Oral hygiene and popular science: Paolo Mantegazza (1831–1910) and the question of sugar in dental caries

**DOI:** 10.1038/s41415-026-9631-9

**Published:** 2026-06-12

**Authors:** Francesco Compagnoni, Michele A. Riva

**Affiliations:** 41415711889001 School of Medicine and Surgery, University of Milano-Bicocca, Italy; 41415711889002 School of Medicine and Surgery, University of Milano-Bicocca; Italy; Department of Occupational Health, Fondazione IRCCS San Gerardo dei Tintori, Monza, Italy

## Abstract

This historical study examines the contribution of the Italian physician and anthropologist Paolo Mantegazza (1831–1910) to the early understanding of the relationship between sugar and dental caries. In his *Elementi d'Igiene* (1864), one of the most influential Italian manuals of public health, Mantegazza conducted a series of laboratory experiments to investigate whether sugar could chemically damage teeth. His results led him to conclude that sugar exerts no direct chemical action on enamel, but that its harmful effects depend on fermentation processes – an interpretation that anticipated, decades in advance, the modern microbiological explanation of caries. This work highlights Mantegazza's pioneering empirical approach, which reflected the emergence of experimental hygiene in 19^th^ century Italy, even in the field of dentistry, and his broader commitment to promoting scientific knowledge as a tool for public education and preventive medicine.

## Key points

- Paolo Mantegazza (1831–1910) was an Italian physician, anthropologist, and populariser of science, whose *Elementi d'Igiene* (1864) became one of the most influential manuals of public health in Italy.
- In his laboratory experiments, Mantegazza demonstrated that sugar exerts no direct chemical action on teeth, anticipating the modern understanding of fermentation as the key mechanism in dental caries.
- His empirical approach reflects the emergence of experimental hygiene in 19^th^ century Italy, including its early applications within the field of dentistry.

## Introduction

That sugar was harmful to the teeth was already a theory circulating since antiquity. The Greek philosopher Aristotle (384–322 BCE) observed that soft and sweet foods such as figs could damage the teeth.^1^ Centuries later, during the Enlightenment, this notion permeated the scientific community. Pierre Fauchard (1678–1761), recognised as the father of modern dentistry, rejected the ancient theory of the ‘tooth worm' and noted that sugar was deleterious both for the teeth and for the gums.^2^ Only in 1890, with the publication of the studies of Willoughby Dayton Miller (1853–1907) on the chemo-parasitic nature of bacteria in the oral cavity and their importance in the initial acid demineralisation of enamel,^1^ did the scientific debate on the aetiology of dental caries reach a consensus. However, it was precisely within this debate that a curious experiment carried out by the Italian physician Paolo Mantegazza (1831–1910) must be placed. In his book *Elementi d'Igiene* (*Elements of Hygiene*, 1864),^3^ among the most influential Italian manuals of public health,^4^ he demonstrated that sugar itself exerts no direct chemical action on the teeth; while nonetheless attempting to explain why it was commonly associated with the development of caries.

## Paolo Mantegazza: physician, anthropologist and scientist

**Fig. 1:**
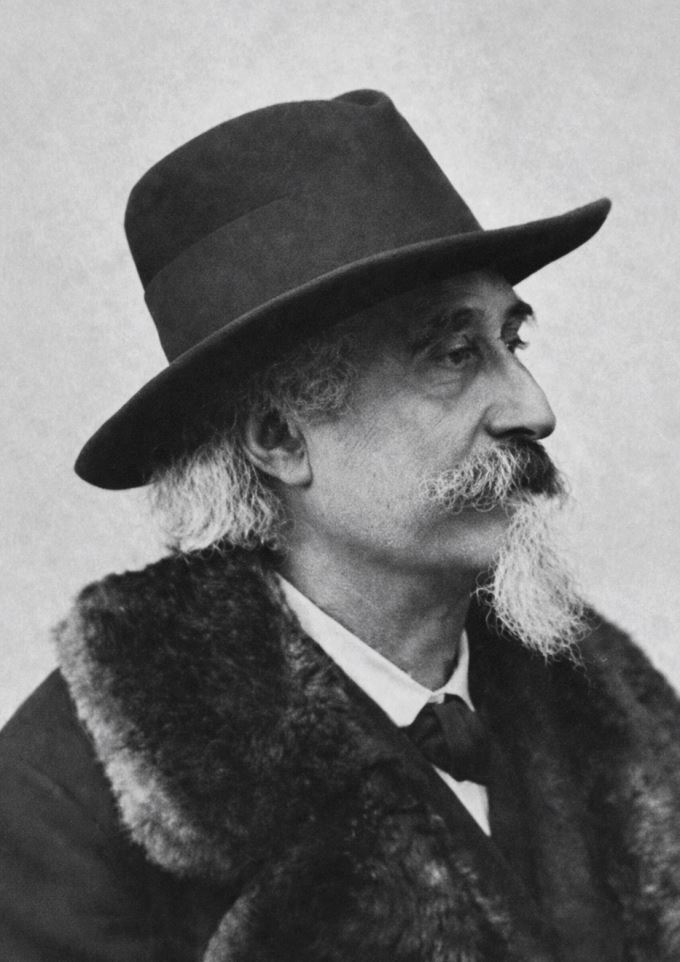
Paolo Mantegazza (1831–1910). Detail from a postcard dedicated to Paolo Mantegazza, from the *‘Italiani illustri'* series curated by Filippo Orlando (1850–1910). Author's personal collection

Paolo Mantegazza (Fig. 1) was born in 1831 in Monza, near Milan, into a family active in the Italian *Risorgimento*. His mother, Laura Solera (1813–1873), was a well-known philanthropist and patriot, remembered for her initiatives in favour of women's education and children's welfare. After completing his medical studies in Pisa and Pavia,^5^ Mantegazza traveled extensively in South America between 1854 and 1858, where he practised as a physician and carried out anthropological and ethnological research.^4^ Upon his return to Italy, he was appointed professor of general pathology at the University of Pavia (1860). There he established one of the first laboratories of experimental pathology in Italy; that institute soon became a fertile training ground for a new generation of Italian biomedical scientists: Giulio Bizzozero (1846–1901), later renowned for his discovery of platelets; Carlo Forlanini (1847–1918), pioneer of the artificial pneumothorax in the treatment of pulmonary tuberculosis; and Camillo Golgi (1843–1926), who went on to receive the Nobel Prize in Physiology or Medicine in 1906 for his studies on the structure of the nervous system.^4^

In 1869 he moved to Florence, where he founded the National Museum of Anthropology and Ethnology and became the first Italian professor of anthropology and ethnology. His intellectual activity was wide-ranging: he investigated the effects of coca leaves, hallucinogens, and other psychoactive substances, becoming a pioneer of psychopharmacology. He was also one of the first to develop a systematic discourse on sexuality with works such as *Fisiologia dell'amore* (*Physiology of Love*, 1873), *Igiene dell'amore* (*Hygiene of Love*, 1878), and *Fisiologia della donna* (*Physiology of Woman*, 1893), which led him to be recognised as a founder of modern sexual medicine.^6^^,^^7^^,^^8^

Mantegazza's career combined science and politics. As a member of the Italian Parliament for more than 20 years, beginning in 1865, first as deputy and later as senator, Mantegazza directed his sociopolitical activity not only toward preventive campaigns against cholera and tuberculosis,^4^ but above all toward the dissemination of medical and scientific knowledge. Inspired by the work of the French anthropologist Paul Broca (1824–1880), he recognised the central importance of popular science for building a better society, founded on the ideals of positivism and social Darwinism.^5^ At the same time, he devoted great effort to science communication, publishing forty volumes of *Almanacchi Igienici Popolari* (*Popular Hygiene Almanacs*, 1865–1905), which became bestsellers across Italy. Through these activities, Mantegazza became one of the most prominent intellectuals of post-unification Italy, embodying the belief that scientific knowledge should serve both as an instrument of progress and as a civic duty. He died on 28 August 1910 in San Terenzo, near La Spezia, where he had spent his final years. His multifaceted legacy – as physician, anthropologist, politician, and scientist – made him one of the most original and influential figures in 19^th^ century Italian culture.

## Testing sugar: Mantegazza's six experiments on dental caries

**Fig. 2:**
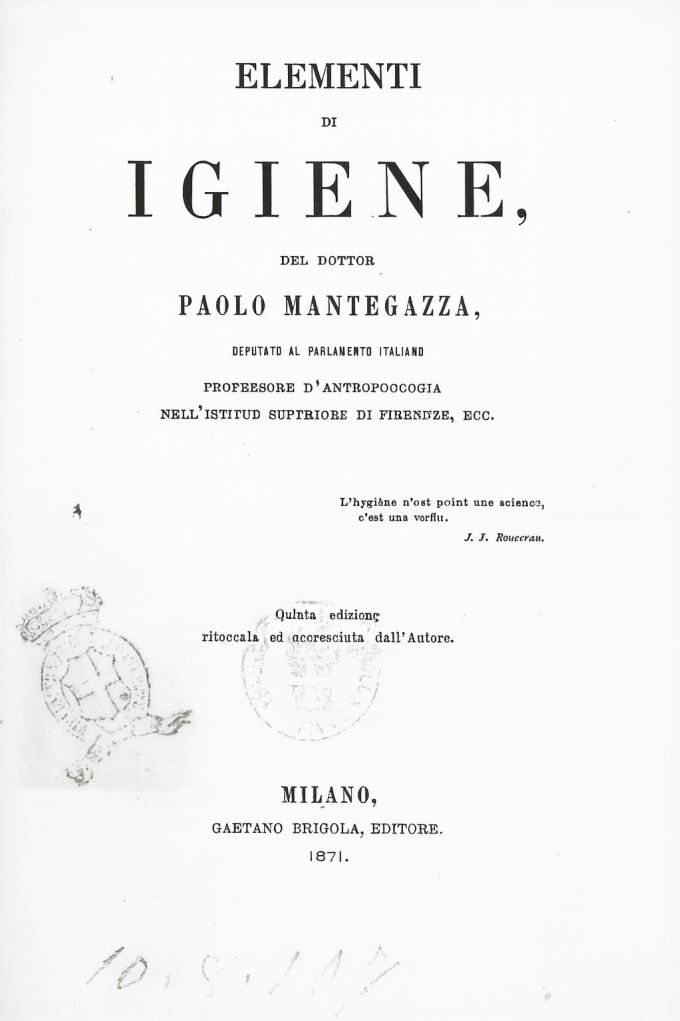
Cover of *Elementi d'Igiene* (1871)

Among Mantegazza's numerous publications, *Elementi d'Igiene* (Fig. 2) occupies a central place. Conceived as a comprehensive manual for both students and cultivated readers, it rapidly became one of the most successful Italian texts of public health, reaching several editions within a short time. The book combined theoretical principles with practical advice, ranging from sleep and diet to exercise and personal hygiene, reflecting the author's ambition to create a ‘science of everyday life'.

Within this broad framework, Mantegazza also addressed the problem of dental caries and the role of sugar. It is precisely within the second chapter of this book – whose topics include ‘Hunger and Thirst […] – On Mastication – Hygiene of the Teeth' – that the author confronted the widespread belief ‘that the use of sugar […] [is] one of the most fertile causes of dental caries'. He observed that the medical community of his time remained silent on the matter: ‘which is why our ignorance finds refuge in the usual compromise of moderation […], use it but do not abuse it'.^3^

Moreover, the confusion surrounding the subject was reflected in the sources cited by Mantegazza, such as *Pathologische Physiologie* (*Pathological Physiology*, 1857) by Gustav Adolph Spiess (1802–1875) and *Volks-Gesundheits-Pflege* (*Care of the People's Health*, 1862) by Eduard Reich (1836–1919). The former asserted that ‘a concentrated solution of sugar can, without needing to become acidic, dissolve and reduce ivory and even enamel into a kind of gelatin,' while the latter stated that ‘sugar cannot cause any harm to healthy teeth'.^3^

Seeking to clarify the issue, Mantegazza, together with an anonymous medical student, organised a series of experiments in his laboratory to verify how, and under what conditions, sugar could in fact affect the structure of teeth.

The experiment consisted of a sequence of ‘experiences,' six in total, in which teeth were taken both from living subjects (‘Teeth are taken from a young peasant woman' – first experience) and from cadavers (‘Ten teeth are extracted from the corpse of a 30-year-old peasant' – third experience), and were then exposed to sugar in various forms and solutions. To assess possible damage to the enamel, Mantegazza used reagents capable of detecting the presence of calcium in the powders or solutions obtained at the end of the different trials, and he also compared the weight of the teeth before and after exposure to the solutions.^3^

In the first experience, some teeth were rubbed with ‘a piece of the purest sugar', producing powders that contained traces of calcium. In the second experiment, however, after keeping a piece of sugar in his mouth for five minutes, the author reported that his saliva contained no trace of calcium, ‘although I have two molars in which the enamel is affected by incipient caries'.^3^

Following these preliminary tests, Mantegazza decided to verify the effect of prolonged exposure of teeth to sugar solutions. Three containers were prepared: two with only water and sugar, the second having twice the concentration of the first, and a third containing saliva and sugar at the same concentration as the first solution. Three groups of teeth were selected and weighed before being placed in the different solutions for thirty days at room temperature.

At the end of the period, the teeth were retrieved and reweighed. The results showed that the teeth with the least weight loss were those immersed in the more concentrated water-and-sugar solution. Mantegazza explained this outcome by writing: ‘the more concentrated sugar solution, which underwent a slower and weaker acid transformation, attacked the teeth half as much as the liquids that, because of their dilution, fermented more easily and produced a greater quantity of acid'.^3^

In the fourth experiment, he repeated the procedure of the third, obtaining the same results. Finally, in the last two experiments, he studied the effects of immersing teeth first in solutions of lactic acid and then in lemon juice or vinegar. The findings were that the lactic acid solution damaged the teeth so strongly and rapidly ‘as to detach pieces of enamel, making the tooth extremely fragile'.^3^ By contrast, the solutions of vinegar or lemon juice corroded the teeth more slowly.

The conclusions that emerged from these simple experiments were that ‘sugar exerts no chemical action on the teeth; it can therefore neither alter them nor predispose them to caries'.^3^ To this he added that sugar might affect teeth ‘mechanically', but in the same way as other hard bodies (a consideration probably introduced to explain the results of the first experiment). Yet the most interesting ideas of his conclusions emerge in the subsequent passages, where Mantegazza wrote that ‘sugar damages teeth only when it has undergone acetic or lactic fermentation'.^3^ This statement does not differ significantly from the actual pathophysiology of dental caries. In fact, it would only be necessary to add the microbiological elements of the process to assemble all the pieces of the puzzle.

It should be recalled, in this regard, that the years in which Mantegazza was writing *Elementi d'Igiene* coincided with those in which Louis Pasteur (1822–1895) was conducting his pioneering research on the fundamental role of microorganisms in fermentation.^1^ Moreover, we must wait until 1884 for Robert Koch (1843–1910) to formulate his famous postulates,^9^ thereby founding modern microbiology, and, as already noted, until 1890 for Willoughby D. Miller to identify the central role of oral microorganisms in dental caries through his chemo-parasitic theory.^10^ Today we know that the process of caries formation can be described as a loss of minerals (demineralisation), which occurs when plaque pH drops below the critical threshold of 5.5.^11^ This acidification is due to a diet rich in sugar and other fermentable carbohydrates that are metabolised into acids by the resident bacteria in dental plaque.^11^ From a modern perspective, it is now well-established that dental caries is a biofilm-mediated disease, in which oral bacteria metabolise fermentable carbohydrates into organic acids that drive enamel demineralisation.^12^ While *Streptococcus mutans* has long been considered a key cariogenic organism, contemporary research emphasises a broader ecological shift in dental plaque driven by frequent sugar intake rather than the action of a single pathogen, thereby completing the microbiological framework that was necessarily absent from Mantegazza's 19^th^ century experiments.^12^

Mantegazza's investigation can be regarded as a pioneering attempt that approximated the correct pathophysiological explanation of dental caries: it was insightful in recognising the role of fermentation, yet it lacked the specificity later provided by microbiological research. Indeed, he concluded the chapter with these final remarks: it is the acid secretion present in the mucus of the mouth that represents the true cause of dental damage, ‘hence the practical usefulness of alkaline tooth powders and especially of vegetable charcoal'.^3^ However, he also admitted that ‘it may be that the abuse of sugar […], contributes to increasing the acidity of the mouth, and thus indirectly acts against the teeth: but this remains to be proven'.^3^

## Conclusion: between hygiene, experiment, and popular science

Paolo Mantegazza occupies a distinctive place in the 19^th^ century debate on sugar and dental caries. His series of experiments, though rudimentary in design and lacking the bacteriological controls that would later become standard, reveal a genuine attempt to approach a common health problem through empirical observation. By excluding a direct chemical action of sugar on dental enamel and emphasising instead the role of fermentation, Mantegazza approximated – albeit in a partial and non-specific manner – the pathophysiological mechanisms that modern microbiology would elucidate only decades later.

At the same time, these experiments exemplify Mantegazza's broader intellectual project. As physician, anthropologist, and populariser, he sought to make science a practical guide for daily life. His *Elementi d'Igiene* and his numerous other writings were not confined to academic circles but addressed a wider audience, contributing to the dissemination of scientific knowledge in post-unification Italy. The case of sugar and caries thus illustrates how Mantegazza combined experimental inquiry with a pedagogical mission, anticipating preventive perspectives in public health.

In retrospect, his investigation may be seen as an emblematic example of 19^th^ century dental hygiene: scientifically limited, yet socially significant. It foreshadowed the microbial revolution in dentistry while at the same time embodying the conviction that science could become a tool for civic progress. 
